# Nucleus-forming jumbophage PhiKZ therapeutically outcompetes non-nucleus-forming jumbophage Callisto

**DOI:** 10.1016/j.isci.2024.109790

**Published:** 2024-04-18

**Authors:** Ampapan Naknaen, Thanadon Samernate, Panida Saeju, Poochit Nonejuie, Vorrapon Chaikeeratisak

**Affiliations:** 1 Department of Biochemistry, Faculty of Science, Chulalongkorn University, Bangkok, Thailand; 2 Institute of Molecular Biosciences, Mahidol University, Nakhon Pathom, Thailand

**Keywords:** Biological sciences, Immunology

## Abstract

With the recent resurgence of phage therapy in modern medicine, jumbophages are currently under the spotlight due to their numerous advantages as anti-infective agents. However, most significant discoveries to date have primarily focused on nucleus-forming jumbophages, not their non-nucleus-forming counterparts. In this study, we compare the biological characteristics exhibited by two genetically diverse jumbophages: 1) the well-studied nucleus-forming jumbophage, PhiKZ; and 2) the newly discovered non-nucleus-forming jumbophage, Callisto. Single-cell infection studies further show that Callisto possesses different replication machinery, resulting in a delay in phage maturation compared to that of PhiKZ. The therapeutic potency of both phages was examined *in vitro* and *in vivo*, demonstrating that PhiKZ holds certain superior characteristics over Callisto. This research sheds light on the importance of the subcellular infection machinery and the organized progeny maturation process, which could potentially provide valuable insight in the future development of jumbophage-based therapeutics.

## Highlights

• The non-nucleus-forming jumbophage Callisto targeting *P. aeruginosa* was discovered
• Replication machinery of Callisto differs from the nucleus-forming jumbophage PhiKZ
• PhiKZ therapeutically outperformed Callisto both *in vitro* and *in vivo* experiments

## Graphical abstract

**Figure:**
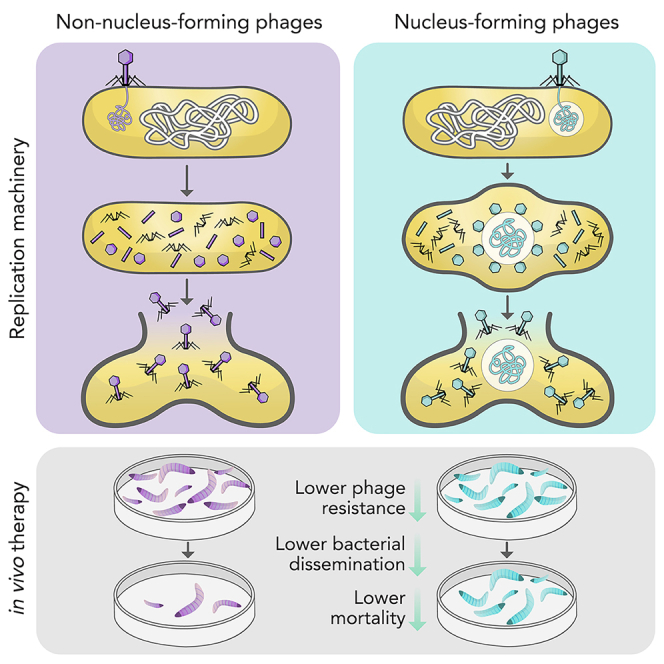

## Introduction

Currently, the state of antimicrobial resistance (AMR) poses a significant worldwide public threat to human health, as indicated by an upward trend in the annual death rate of bacterial infections. According to the 2019 study, approximately 1.27 million fatalities are caused by AMR infection, and it is projected that by 2050, the mortality rate might increase to 10 million.^1^ The World Health Organization (WHO) recently identified some bacterial species that are extremely worrisome and require extensive monitoring. In particular, the prevalence of multi-drug resistant (MDR) strains of *Enterococcus* spp., *Staphylococcus aureus*, *Klebsiella* spp., *Acinetobacter baumannii*, *Pseudomonas aeruginosa*, and *Enterobacter* spp., together known as "ESKAPE," is a major cause for concern.^2^ Notably, *P. aeruginosa*, an opportunistic bacterium that leads to bloodstream infections, pneumonia, urinary tract infections, and surgical site infections, is one of the most significant hospital-acquired bacterial infections. Epidemiological studies have reported an extensive increase in MDR *P. aeruginosa* in nosocomial infections and in patients.^3^^,^^4^^,^^5^ This is partly due to the fact that the bacteria are capable of enduring harsh conditions in hospitals and hosts by releasing many virulence factors, including a self-protective biofilm.^6^ Additionally, this pathogen demonstrates a remarkable capacity to develop antibiotic resistance in people who are hospitalized.^7^^,^^8^^,^^9^

Although there is a growing need for novel antibiotics to combat AMR, the development of new antimicrobial agents is declining,^10^ suggesting that the overall process of antibiotic discovery is currently insufficient to match the rapid pace of AMR. Moreover, several antibiotics currently undergoing clinical trials are modified versions of existing medications, making them susceptible to drug resistance mechanisms in bacteria that have developed from the same chemical structure.^1^^,^^11^ Although the discovery of Zosurabalpin,^12^ a new class of antibiotics that target bacterial LPS transport, has sparked significant optimism in the scientific community for its potential to combat drug-resistant bacteria such as Carbapenem-resistant *A. baumannii* (Crab), *Enterobacteriaceae*, and *P. aeruginosa*, the advancement of these new drugs through the development pipeline may face challenges.^13^ Considering anticipated difficulties regarding discovering new antibiotics, it is crucial to pursue alternate treatments for bacterial infections, such as antimicrobial peptides,^14^ anti-virulence compounds,^15^ and notably, bacteriophage therapy.

Bacteriophage therapy has been increasingly recognized as a possible therapeutic approach^16^^,^^17^ to combat MDR bacteria.^18^ Bacteriophage, also known as phage, is considered a living antibiotic because it has the ability to selectively kill a particular kind of bacteria at the sites of infection, making it possess superior characteristics over traditional antibiotics.^19^ Phage therapy has demonstrated global potential in terms of both efficacy and safety for the treatment of human infections, drawing greater attention in recent clinical studies, particularly for treating *P. aeruginosa* infections.^6^^,^^17^ Despite its efficacy, one of the limitations of phage therapy is the rapid development of bacterial resistance to phage infection.^20^ Bacteria employ numerous strategies that hinder phage infection,^21^ including surface alterations, superinfection exclusion (Sie), and defensive systems linked to clustered regularly interspaced short palindromic repeats (CRISPR)-Cas,^22^ which undermine the efficacy of phage therapy.^21^^,^^23^ To overcome phage resistance, the use of a phage cocktail is commonly employed to combat resistance.^24^ Nonetheless, it is also important to choose candidate phages that possess distinct characteristics (i.e., bacterial immune counteract capabilities and unique replication machinery) in order to develop an effective phage therapy regimen.^21^^,^^22^ For example, jumbophages capable of assembling a nucleus that effectively protects their DNA against host CRISPR nucleases^25^^,^^26^ would be one of the potential strategies to counteract bacterial defense mechanisms, thereby mitigating phage resistance in bacteria.

Jumbophages, which possess a genome size over 200 kb,^27^ have been historically neglected, partly because of the traditional criteria used to assess the effectiveness of phages as therapeutic agents. These criteria primarily emphasize factors such as adsorption rate, latent period, and burst size, which are areas where jumbophages tend to underperform compared to other small phages.^27^^,^^28^ Nevertheless, researchers have become increasingly intrigued with jumbophages in recent years. This is not only because of their ability to shield the genome from the bacterial host defense mechanisms mentioned earlier,^25^^,^^26^ but also because of fascinating aspects of their biology.^27^^,^^28^ These characteristics include larger phage virions, diverse propagation mechanisms, and the existence of subcellular compartment systems.^25^^,^^26^^,^^29^^,^^30^^,^^31^^,^^32^^,^^33^^,^^34^^,^^35^^,^^36^ Furthermore, due to their large genomes, the jumbophages possess various gene products that can substitute some host proteins necessary for the phage life cycle. This minimizes the jumbophages’ dependence on host organisms for replication,^7^ resulting in a wider host range compared to smaller phages. The use of a jumbophage cocktail has been recently proven successful as it effectively manages the contamination of extensively drug-resistant *P. aeruginosa* in preservative-free eyedrops in the United States.^37^ The application of a jumbophage cocktail is not only limited to clinical use; it can also be utilized as a biocontrol in aquaculture.^38^ Collectively, these distinctive characteristics underline the jumbophages' capacity as potential candidates for therapeutic applications.

However, it should be noted that not all jumbophages are created equally and are appropriate for therapy. Up to date, comprehensive studies of jumbophage evolution have been elusive, not only due to the limited number of available jumbophages in the database but also their highly diverse genomes,^27^ rendering it difficult to classify individuals into certain groups. Despite the lack of rigid classification frameworks, the jumbophages could be primarily classified based on the presence of some conserved core genes as PhiKZ-like phages (recently reclassified into the *Chimalliviridae* family) according to the presence of the conserved core genes involved in nucleus-based replication, T4-like and T7-like viruses, due to the presence of conserved T4 DNA helicase and T7 DNA polymerase, respectively.^27^^,^^28^^,^^30^ Additionally, based on a gene-sharing based-analysis, the majority of jumbophages are clustered into 2 major viral clusters: PhiKZ-like and T4-like viruses, with a few unclassified.^27^^,^^30^ While significant work regarding fundamental and therapeutic properties has been achieved for the nucleus-forming PhiKZ-like jumbophages,^25^^,^^26^^,^^29^^,^^31^^,^^33^^,^^34^^,^^36^^,^^39^ comprehensive knowledge of the biological features of T-like jumbophages is still lacking, especially their therapeutic potential. Here, as we successfully isolated a jumbophage named Callisto, which was closely related to T4-like viruses, we sought to compare it with the well-studied nucleus-forming jumbophage PhiKZ in various aspects, including genomic organization, replication machinery as well as *in vitro* and *in vivo* therapeutic potential to explore its potential for jumbophage-based therapy.

## Results

### Phage Callisto morphological and biological characteristics

**Figure 1:**
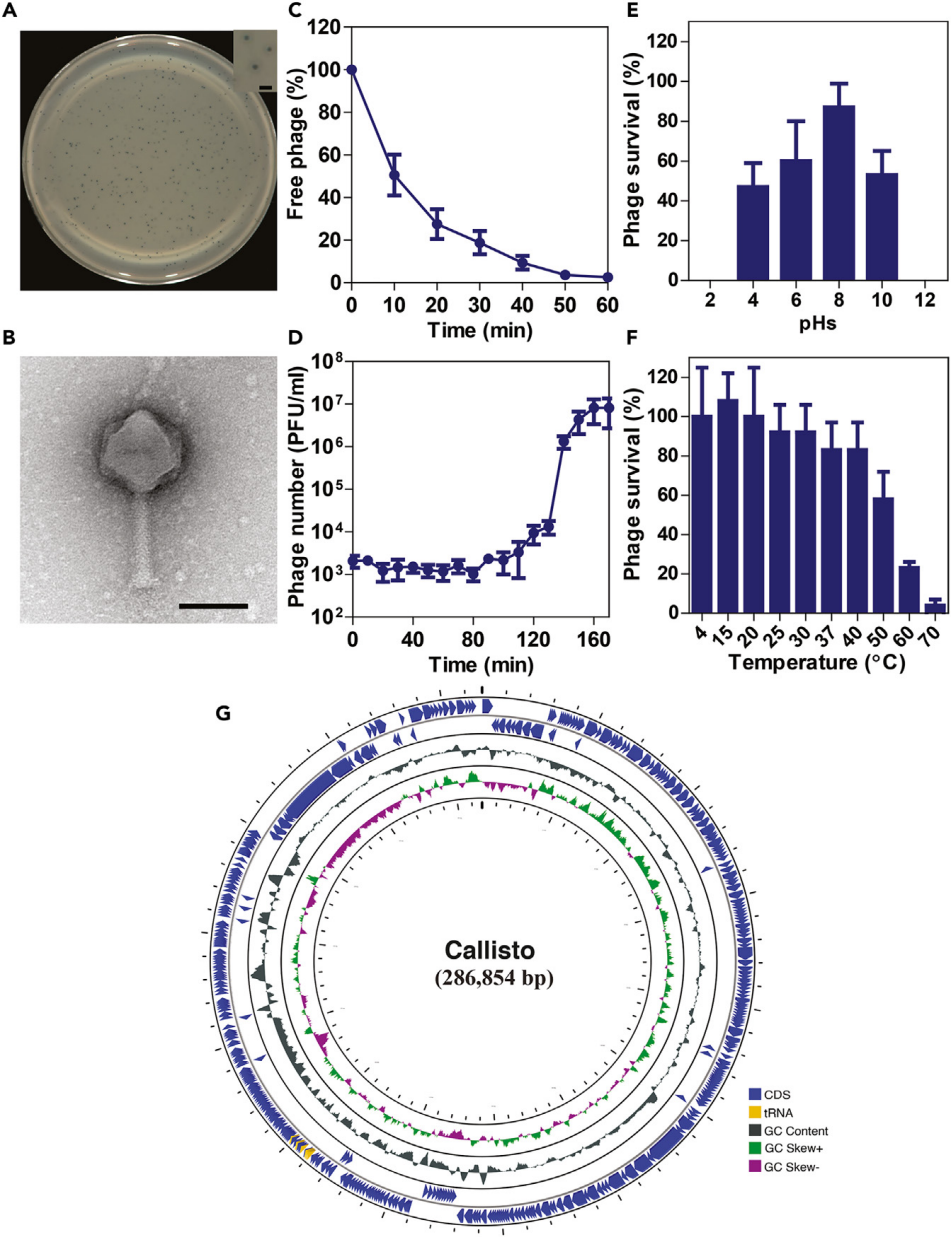
Morphological, biological and genomic characteristics of phage Callisto (A) Plaques of phage Callisto formed on *P. aeruginosa* PAO1 cell lawn. Individual plaques are enlarged as shown in a subpanel. Scale bar equals 1 mm. (B) Electron micrograph of phage Callisto negatively stained with 2% uranyl acetate. The scale bar equals 100 nm. Biological properties of phage Callisto; Adsorption rate (C), One-step growth curve (D), phage stability in different pHs (E) and temperatures (F) for 6 h. The data in (C-F) represent the mean ± standard deviation of at least 3 independent biological replicates. (G) Schematic genome maps of phage Callisto. The innermost circles colored in green, and purple indicate the positive and negative GC skew, respectively. The open reading frames (ORFs) and tRNA are indicated in blue and yellow colors, respectively, with arrows indicating the ORF direction (See also Table S1).

The phage Callisto was isolated from a reservoir at Chulalongkorn University, Bangkok, Thailand (coordinates on a map: 13.73804, 100.53183), using *P. aeruginosa* PAO1 as the parental bacterial host. It was capable of forming a very small, clear plaque with a diameter around 0.35 ± 0.15 mm (*n* = 30) on the cell lawn of the parental host (Figure 1A). Transmission electron microscopy (TEM) revealed that phage Callisto had an icosahedral head (height: 103 ± 3 nm, *n* = 11; width: 102 ± 3 nm, *n* = 11) and a long contractile tail (height: 119 ± 14 nm, *n* = 11; width: 10 ± 1 nm, *n* = 11) (Figure 1B). This phage morphology can be classified into the *Myoviridae* family according to the criteria proposed by Ackermann.^40^

To first explore the biological characteristics of the phage Callisto, we investigated the rate of phage attachment to bacterial cells by adsorption assay, the phage replication cycle in the host cells by a single-step growth curve, and the phage tolerance. The results demonstrated that the majority of phage Callisto (∼85%) adsorbed onto the host cells within 30 min post-infection (mpi) (Figure 1C), and then the phage underwent subcellular activity to replicate in the host cells for at least 100 min. At the end of the latent period, phage Callisto later burst the host cell for phage egress, resulting in estimated burst sizes of approximately 70 phage particles per infected cell (Figure 1D). Phage Callisto appeared to be tolerant of a wide range of pHs and temperatures. The phage was viable at pHs from 4 to 10 but completely lost its infectivity at extreme acidic or alkaline conditions (Figure 1E). Also, the phage was stable and remained infectious at temperatures from 4°C to 40°C and partially ineffective at 50°C (∼60% infectivity). However, the phage was almost completely inactive when the temperature was above 60°C (Figure 1F).

To further explore the potential of phage Callisto for medical and biotechnological applications, we tested the host spectrum, investigated the efficiency of plating (EOP) of the phage against lab strains and clinical strains of *Pseudomonas* spp.^41^^,^^42^ and examined the presence of unwanted genes in the phage genome associated with bacterial virulence, antibiotic resistance, and the lysogenic life cycle that are disadvantageous for phage therapy. The results revealed that phage Callisto had a relatively broad host range compared to phage PhiKZ,^41^ as indicated by its potential to form a clear zone in approximately half of the *Pseudomonas* spp. tested (11/23) (Table 1). Rather than infecting the lab strain of *P. aeruginosa*, it was able to infect 8 clinical strains of *P. aeruginosa*: highly productive in clinical strain PSU-PA05, medium productive in clinical strains PSU-PA06 and PSU-PA08, low productive in clinical strain PSU-PA09, and inefficient in clinical strains PSU-PA04, PSU-PA07, PSU-PA010, and PSU-PA14 (Table 1), indicating its potential in treatment in the clinical setting. It is not surprising that phage Callisto productivity is inefficient in some clinical strains. Since lipopolysaccharide (LPS) O-antigen on the bacterial cell surface, which is a potential receptor required for phage Callisto entry,^43^ is so diverse in *P. aeruginosa* strains,^44^ some modifications or loss of O-antigen in our clinical isolates might significantly affect the phage Callisto activity, as also suggested by previous studies.^45^^,^^46^ At the genomic level, phage Callisto contained a 286,854-bp genome with a GC content of 33.3% and was thereby classified as a jumbophage according to its genome size, which was larger than 200 kb.^27^ Among annotated genes in the phage genome, there were no antibiotic resistance, virulence, or lysogeny-related genes such as integrase, excisionase, or repressor genes (Figure 1G; Table S1). Phage AI analysis also identified phage Callisto as a lytic phage based on genome-based prediction, further supporting the safety of its use.^47^ Altogether, due to its capability to target various clinical *P. aeruginosa* isolates, its tolerance over a wide range of pHs and temperatures, and the absence of undesired genes within the phage genome, phage Callisto is considered appropriate for use in therapeutics and biotechnological purposes.

**Table 1:** Bactericidal spectrum and efficiency of plating (EOP) of phage Callisto

*Pseudomonas* spp.	Source	EOP value
*P. aeruginosa* PAO1	Lab strains	1.000
*P. aeruginosa* PSU-PA01	Clinical strain, Rectal swab	–
*P. aeruginosa* PSU-PA02	Clinical strain, Rectal swab	–
*P. aeruginosa* PSU-PA03	Clinical strain, Rectal swab	–
*P. aeruginosa* PSU-PA04	Clinical strain, Throat swab	≤0.001
*P. aeruginosa* PSU-PA05	Clinical strain, Rectal swab	1.500
*P. aeruginosa* PSU-PA06	Clinical strain, Rectal swab	0.120
*P. aeruginosa* PSU-PA07	Clinical strain, Throat swab	≤0.001
*P. aeruginosa* PSU-PA08	Clinical strain, Throat swab	0.130
*P. aeruginosa* PSU-PA09	Clinical strain, Rectal swab	0.019
*P. aeruginosa* PSU-PA10	Clinical strain, Throat swab	≤0.001
*P. aeruginosa* PSU-PA11	Clinical strain, Throat swab	–
*P. aeruginosa* PSU-PA13	Clinical strain, Throat swab	–
*P. aeruginosa* PSU-PA14	Clinical strain, Throat swab	≤0.001
*P. aeruginosa* ATCC9027	American Type Culture Collection	≤0.001
*P. aeruginosa* ATCC15442	American Type Culture Collection	≤0.001
*P. aeruginosa* ATCC27853	American Type Culture Collection	–
*P. stutzeri* DMST28410	Department of Medical Sciences, Thailand	–
*P. stutzeri* DMST12562	Department of Medical Sciences, Thailand	–
*P. mendocina* ATCC25411	American Type Culture Collection	–
*P. fluorescens* ATCC13525	American Type Culture Collection	–
*P. putida* ATCC12633	American Type Culture Collection	–
*P. putida* ATCC17522	American Type Culture Collection	–

EOP values were classified as highly productive (≥0.5), medium productive (0.1 ≤ EOP < 0.5), low productive (0.001 < EOP < 0.1) or inefficient (≤0.001).

### Genomic study of jumbophage Callisto reveals genetic divergence from nucleus-forming jumbophages

Phage Callisto was a jumbophage containing a large genome, encoding 402 open reading frames (ORFs) and 12 tRNAs (Figure 1G; Table S1). While the majority (∼86%) of ORFs encoded hypothetical proteins with unknown functions, 56 ORFs were functionally annotated by the PHASTER, RAST, GeneMark, and GenBank databases (Table S1). The annotated proteins were involved in DNA replication, including DNA primase (ORF36), ATP-dependent helicase (ORF42), single-stranded DNA binding protein (ORF48), RecA-like protein (ORF51), DNA primase-helicase subunit (ORF65), DNA adenine methyltransferase (ORF92), DNA ligase (ORF108), DNA polymerase III (ORF111), ATP-dependent helicase (ORF159), RNase H (ORF167), deoxi-UTP pyrophosphatase (ORF168), and putative DNA polymerase (ORF394). At least three predicted enzymes were involved in nucleotide metabolism, including endonuclease subunit D12 (ORF38), single-stranded DNA-specific exonuclease (ORF59), and holliday junction ATP-dependent DNA helicase RuvB (ORF113). However, phage Callisto did not harbor RNA polymerases in the genome, suggesting that RNA transcription in the phage is host-dependent. The phage Callisto structural proteins included those components in the virion structure such as major capsid proteins (ORF37 and ORF41), tail fibers (ORF1, ORF7, ORF49, ORF55, ORF56, and ORF81), virion structural proteins (ORF292 and ORF300), and a DNA packaging protein (ORF154; HNH homing endonuclease III). Phage Callisto also encoded the baseplate hub subunit and tail lysozyme (ORF86) that were associated with peptidoglycan layer degradation.

**Figure 2:**
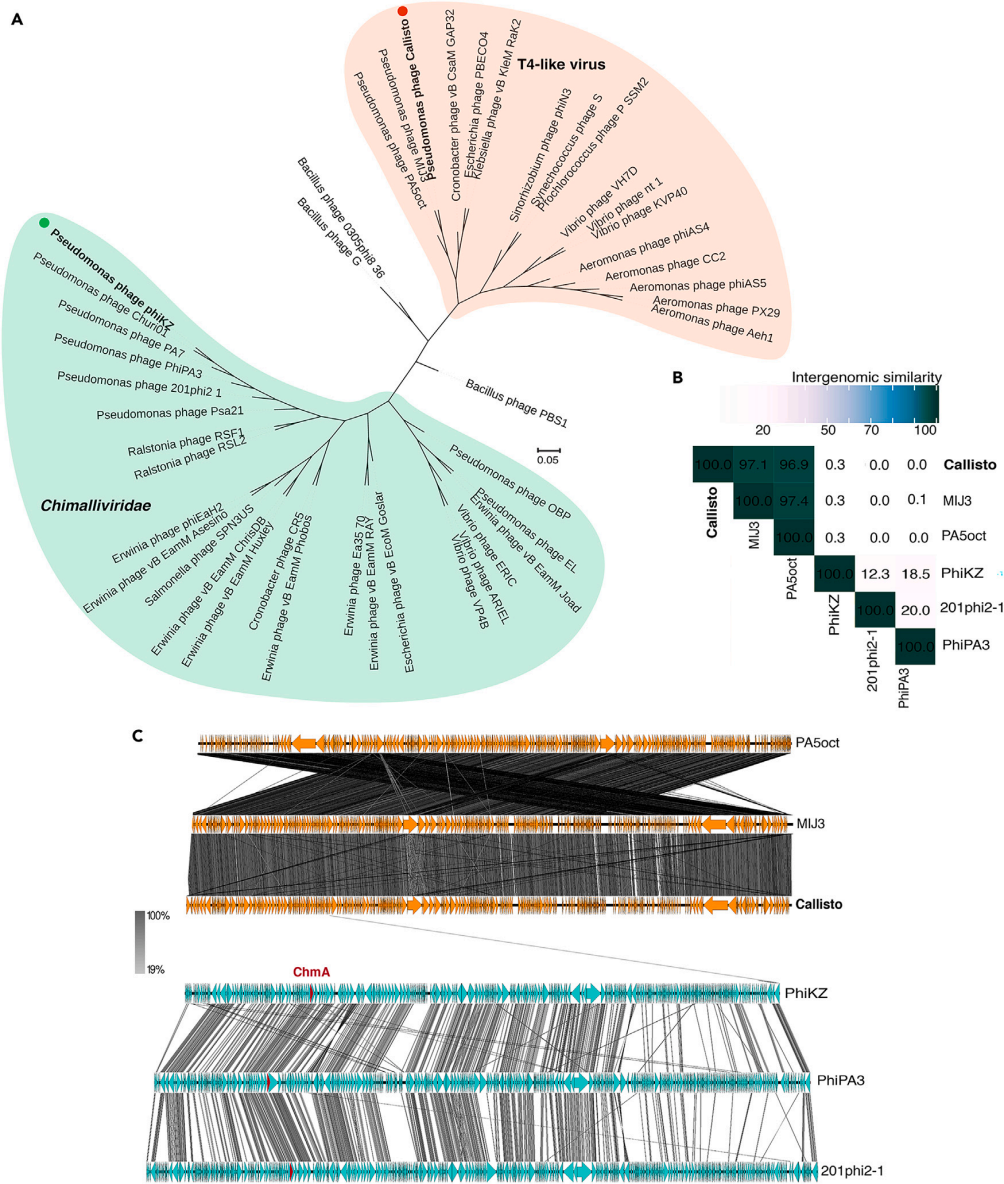
Jumbophage Callisto is clustered with T4-like jumbophages and is genetically diverse from nucleus-forming jumbophages in the *Chimalliviridae* family (A) A whole-genome sequence-based phylogenetic tree of jumbophage Callisto (orange dot) and other selected jumbophages. The tree was constructed using the Genome-BLAST Distance Phylogeny (GBDP) method and generated with VICTOR web service with 100 bootstraps, followed by visualization via iTOL (*Chimalliviridae*; green, and T4-like jumbophages; orange). (B) VIRIDIC heatmap showing the intergenomic similarities of selected T4-like viruses (Callisto, MIJ3, and PA5oct) and the well-studied PhiKZ-like viruses (PhiKZ, PhiPA3, and 201Phi2-1). The numbers represent the similarity values for each genome pair. (C) Comparative genome analysis of jumbophage genomes; T4-like viruses (Callisto, MIJ3, PA5oct; orange) and the well-studied PhiKZ-like viruses (PhiKZ, PhiPA3, and 201Phi2-1; green). The arrows represent the directions and locations of coding sequences. Gray-shaded lines reflect the degree of homology between them.

Currently, based on morphological similarity and genomic relationships, the jumbophages can be mainly divided into two major groups: the T-like viruses and the chimalliviruses.^48^^,^^49^^,^^50^ To date, the presence of conserved core genes involved in nucleus-based phage replication is one of the solid genetic evidences used to classify nucleus-forming jumbophages in the *Chimalliviridae* family.^30^ During infection, chimalliviruses assemble a nucleus-like structure called “the phage nucleus,” where the phage genome replicates within and is protected from host defense. The phage nucleus is mostly made of the conserved chimallin A protein (ChmA) and serves various crucial roles during the lytic life cycle of the chimalliviruses. To investigate the evolutionary relationship of our jumbophage Callisto with these nucleus-forming jumbophages, we constructed a phylogenetic tree using the Genome-BLAST Distance Phylogeny (GBDP) method by the VICTOR web service among selected jumbophages from the viral cluster of T4-like jumbophages as analyzed by gene-sharing network^48^ and the chimalliviruses^30^ with *Bacillus* phage PBS1 as an outgroup. The tree revealed that jumbophage Callisto (Figure 2A; orange dot) was clustered within the clade of T4-like viruses and was closely related to *Pseudomonas* phages MIJ3 and PA5oct (Figure 2A; orange shade). The T4 clade was completely separated from the *Chimalliviridae* clade (Figure 2A; orange and green shades), suggesting the high genetic variation between these jumbophage families. To further examine the genetic relationship and compare the genome organization of jumbophage Callisto to the closely related phages MIJ3 and PA5oct and the well-studied chimalliviruses PhiKZ, PhiPA3, and 201Phi2-1, we performed comparative genomic analyses among these jumbophages. The result confirmed the evolutionarily close relationship of phage Callisto to phages MIJ3 and PA5oct, revealing ∼97% intergenomic similarity of phage Callisto to the T4-like viruses, phages MIJ3^51^ and PA5oct,^48^ with a similar genome organization to phage MIJ3 (Figures 2B and 2C). However, jumbophage Callisto shared very low intergenomic similarity (0–0.3%) to the chimalliviruses PhiKZ, PhiPA3, and 201Phi2-1 (Figure 2B). As further illustrated by the genome comparison, jumbophage Callisto organized its genome largely differently from the chimalliviruses. Its genome did not harbor any conserved homologs to the chimalliviruses, including the chimallin A protein (ChmA), except for one conserved ribonucleotide reductase (Figure 2C). Altogether, these results suggest that jumbophage Callisto is a new member of T4-like viruses belonging to the *Tevenvirinae* and genetically diverged from nucleus-forming jumbophages.

### The replication machinery of jumbophage Callisto is mechanistically different from that of nucleus-forming jumbophage PhiKZ

**Figure 3:**
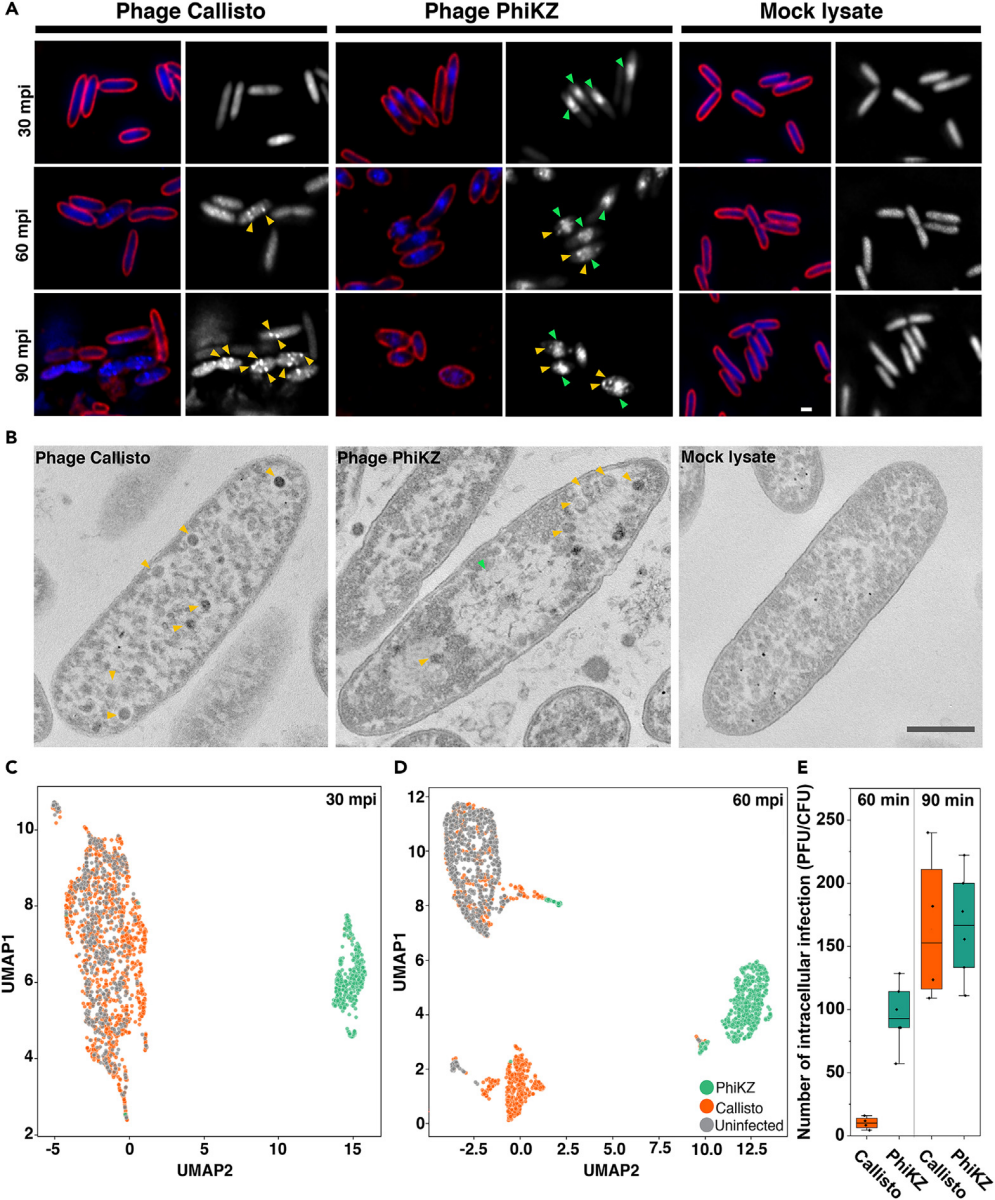
Jumbophage Callisto exhibits different replication machinery during infection from nucleus-forming jumbophage PhiKZ at the single-cell level (A) Time-series images over a course of 90 min of *P. aeruginosa* treated with either phage Callisto (left panel), phage PhiKZ (middle panel), or mock lysate (control; right panel). *P. aeruginosa* cells at OD_600_ ∼ 0.4 were infected with the indicated phage at MOI 10 and later fixed at 30-, 60-, and 90-min post-infection (mpi). The cells were stained with FM4–64 (cell membrane; red) and DAPI (nucleoid; blue or gray). Scale bar equals 1 micron. (B) Conventional ultrathin sectioning and transmission electron microscopy images of *P. aeruginosa* treated with either Callisto (left panel), PhiKZ (middle panel), or mock lysate (right panel) at 60 mpi. In phage-infected cells, icosahedral phage particles (yellow arrows) assembled and appeared in the host cell. The presence of the phage nucleus was observed in PhiKZ-infected cells (green arrows). Scale bar equals 500 nm. (See also Figure S1). (C and D) The cytological profile was performed by Uniform Manifold Approximation and Projection (UMAP), showing cell clusters of uninfected cells (gray), cells infected with Callisto (orange) and PhiKZ (green) at 30 mpi (C) and 60 mpi (D). (E) Intracellular progenies of Callisto (orange) and PhiKZ (green) at late time points. The experiment was performed in at least independent triplicates and the data represent the mean ± standard deviation.

Based on the distant genetic variation of jumbophage Callisto from the nucleus-forming jumbophages or chimalliviruses, we hypothesized that the replication machinery of jumbophage Callisto that occurs inside the host cells during the lytic life cycle might be largely different from that of the nucleus-forming jumbophages. To observe the progress of the replication cycle of the phage Callisto in comparison to the well-studied chimalliviruses PhiKZ, we performed a single-cell infection assay and observed DAPI-staining DNA to follow the phage reproduction process in the host cells.^41^^,^^52^ The results showed that, during the phage infections, the infected *Pseudomonas* cells had their morphology altered throughout the time, which might be a result of the phage infections compared to the uninfected cell control (Figures 3A, 3C, and 3D). During infection with phage PhiKZ (Figure 3A; PhiKZ), the phage assembled the phage nucleus localized close to the midcell of the host (Figure 3A; PhiKZ, green arrows). As the infection proceeded, many small puncta appeared close to the surface of the phage nucleus at 60 mpi, later detached from the nucleus, and localized in the cytoplasm of the host cells at 90 mpi (Figure 3A; PhiKZ, yellow arrows). These observations corresponded to what was previously shown in our studies,^33^^,^^34^ indicating the progression of phage PhiKZ maturation. Since these small puncta are mature capsids that have packaged the phage genome (Figures 3A and 3B; PhiKZ, yellow arrows),^35^ we acknowledge the presence of these foci as a hallmark of the completion of DNA encapsidation. In comparison to phage PhiKZ infection, during the infection of phage Callisto at 30 mpi, the phage did not assemble any DNA-containing structures and did not trigger any morphological changes in the host (Figures 3A and 3C). However, at late stages of infection, many DAPI-staining puncta uniformly appeared throughout the infected cells at 60 mpi and seemed to increase in number at 90 mpi (Figures 3A and 3D).

To further confirm whether these DAPI-staining puncta are truly DNA-packaged capsids of phage Callisto, we conducted conventional ultrathin sectioning and TEM to observe the phage Callisto-infected cell at high resolution. The result revealed icosahedral dark particles with a diameter of around 100 nm that were distributed throughout infected cells (Figure 3B; phage Callisto and yellow arrows; Figure S1). This observation agrees well with the result of DAPI-staining puncta as observed by fluorescence microscopy (Figures 3A and 3B; phage Callisto and yellow arrows; Figure S1), thereby suggesting that, unlike nucleus-forming jumbophage PhiKZ, in which phage replication and maturation are highly organized in a nucleus-based manner, the genome replication and mature capsid assembly of phage Callisto occur independently in multiple unique viral replisomes (Figures S1C–S1E). Moreover, the phage particles of phage Callisto at this late stage were infectious virions. As quantitated in Figure 3E, the number of intracellular viral progeny at 60 mpi was around 10 virions per cell. Even though the viral progeny number in phage Callisto was ∼10 times lower than that in phage PhiKZ at 60 mpi, the number of progenies was at a comparable level at 90 mpi (Figure 3E).

One of the well-known features of PhiKZ-like viruses is that their transcriptional mechanism is solely dependent on their own multisubunit RNA polymerase (RNAPs).^53^^,^^54^^,^^55^ After careful analysis of all proteins in the phage Callisto genome, however, no virion-associated RNAPs could be detected. To further demonstrate whether the transcription of phage Callisto depends on the host RNAP, rifampicin (RIF) was used as an inhibitor for the host RNA polymerase. Prior to infection with phages, the bacterial cells were treated with RIF (100 and 400 μg/mL) for 30 min at 37°C. The results showed that RIF completely blocked the replication of phage Callisto since none of the DAPI-staining puncta appeared in the infected cells and the intracellular viral progenies were not detected (Figure S2). However, PhiKZ, which is independent of the host transcriptional machinery, was capable of producing a comparable number of viral progenies in the RIF-treated conditions to the untreated cell control (Figure S2). Altogether, these data corresponded to the evolutionary divergence between phages Callisto and PhiKZ and further demonstrated that the replication machinery between non-nucleus-forming jumbophage Callisto and nucleus-forming jumbophage PhiKZ is mechanistically different.

### The nucleus-forming jumbophage PhiKZ outcompetes the non-nucleus-forming jumbophage Callisto in both killing activity *in vitro* and therapeutic effect *in vivo*

**Figure 4:**
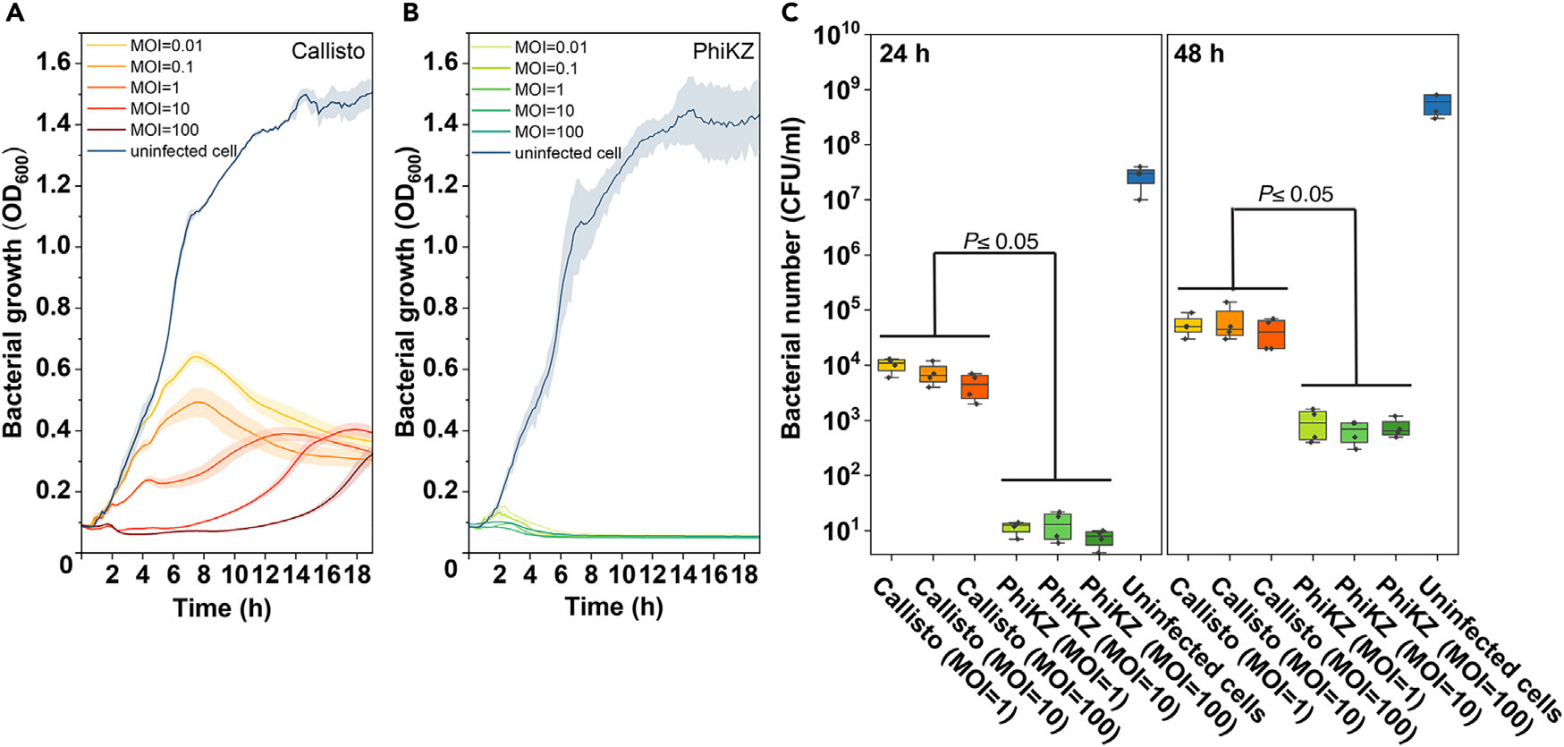
The nucleus-forming jumbophage PhiKZ effectively suppresses bacterial growth and reduces the phage resistance compared to the non-nucleus-forming jumbophage Callisto The killing kinetics of jumbophages Callisto (A) and PhiKZ (B) at MOI ranging from 0.01 to 100 over a window of 18 h. (C) The revival of bacteria after the treatment with either Callisto or PhiKZ at MOIs of 1, 10, and 100 at 24 h and 48 h. The data represent the mean ± standard deviation of at least 3 independent biological replicates. Statistical significance in (C) was calculated using two-way ANOVA with Tukey’s HSD test.

One of the benefits of nucleus-based phage replication is a strategy to evade bacterial defenses by excluding DNA-targeting Cas enzymes and restriction enzymes.^25^^,^^26^^,^^29^ Based on the aforementioned benefit, together with the highly organized replication factory of PhiKZ-like viruses, we reasoned that the nucleus-forming jumbophages might display more advantages over the non-nucleus-forming jumbophages. We therefore investigated the killing activity *in vitro* and therapeutic effect *in vivo* in the non-nucleus-forming jumbophage Callisto to compare them with those in the nucleus-forming jumbophage PhiKZ. To examine the killing efficacy of phages Callisto and PhiKZ, we first followed the bacterial density in the presence of the phage for 18 h at various MOIs from 0.01 to 100. The results showed that the growth of the bacterial host treated with phage Callisto at low MOIs of 0.01 and 0.1 was not well suppressed in the early time frame. The bacterial density increased continuously until reaching ∼7 h of incubation, followed by a declining trend during the rest of the experiment. At higher MOIs (10 and 100), the bacterial growth was better suppressed from the beginning to ∼11 h of incubation and increased continuously until the end of the experiment (Figure 4A). The killing kinetics of phage Callisto indicated that the phage inhibited bacterial growth in a dose-dependent manner and that bacterial resistance might emerge after the treatment. Surprisingly, bacterial growth was completely suppressed by the phage PhiKZ at all tested MOIs throughout the test period (Figure 4B). Even though, at the longer time of treatment with phage PhiKZ (24 h and 48 h), the viable bacterial number can still be detected (Figure 4C), these viable bacteria in the presence of phage PhiKZ still remained 2–3 log lower than those in the presence of phage Callisto (Figure 4C). Altogether, these findings suggest the *in vitro* efficient activities of nucleus-forming jumbophage PhiKZ in both suppressing bacterial regrowth and minimizing the emergence of phage-resistant bacteria.

**Figure 5:**
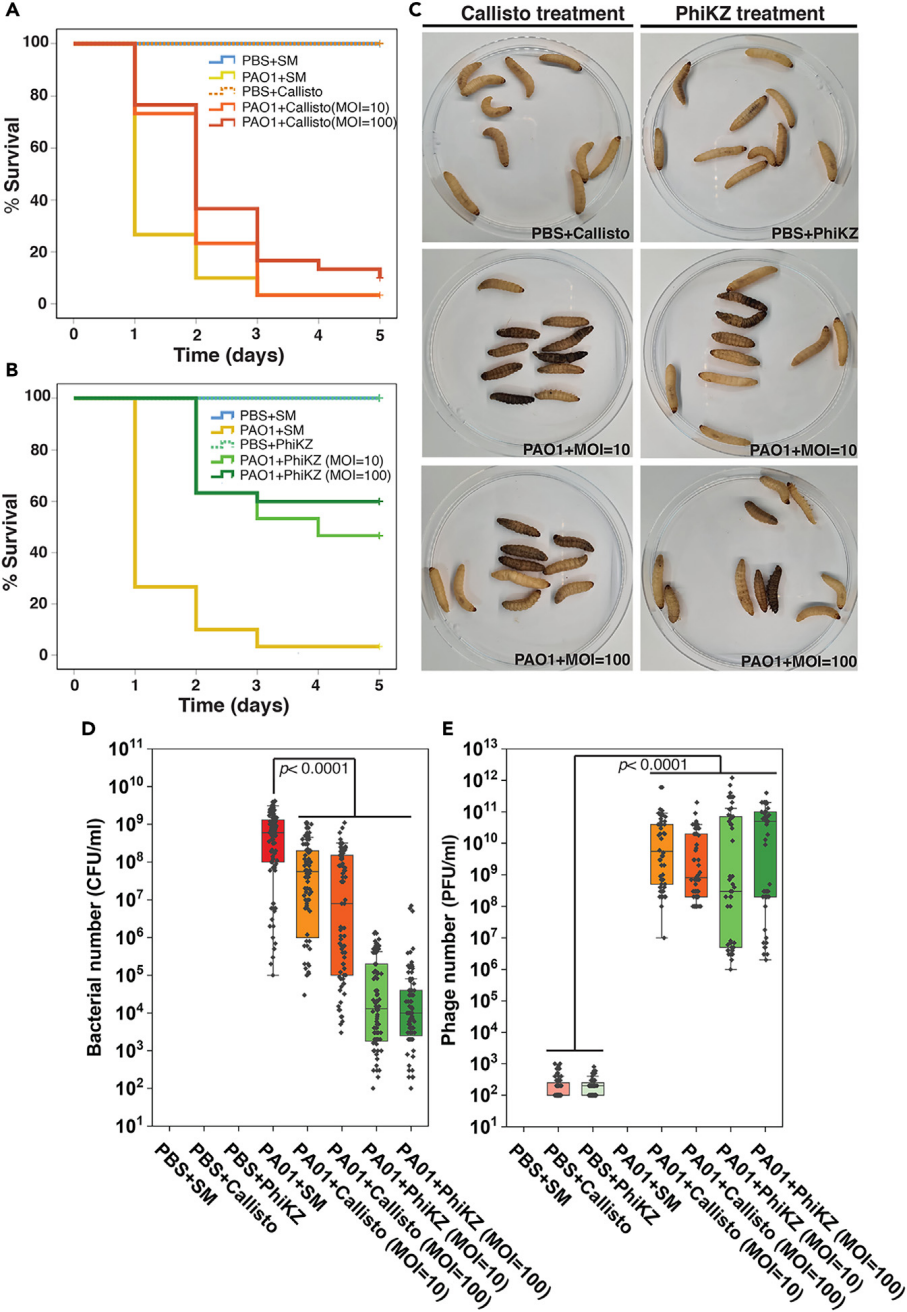
The nucleus-forming jumbophage PhiKZ therapeutically outcompetes the non-nucleus-forming jumbophage Callisto in *G. mellonella* model (A and B) The survival percentage of the larvae infected with PAO1, followed by the treatment with either phages Callisto (A) or PhiKZ (B). The phage at MOIs of 10 and 100 was injected after the larvae were pre-infected with PAO1 for 1 h (*n* = 10 per group). The mortality of infected animals was monitored for 5 days. The group injected with PBS and SM buffer was used as the negative control whereas the group injected with PAO1 and SM buffer was used as the positive control. The experiment was conducted in three independent replicates. The survival rates were plotted using the Kaplan-Meier method in SPSS. (C) Larvae infected with PAO1, followed by the treatment with either phages Callisto or PhiKZ. The larvae appearing black (melanization) or no movement were considered as dead and the larvae that showed movement without stimulation or no melanization were considered as alive. (See also Figure S3) (D) Viable *P. aeruginosa* remaining in the larvae of all groups on day 5 represented as CFU/ml. (E) Viable phage number from larvae of all groups on day 5 represented as PFU/ml. n equals to 30 larvae per group. The data represent the mean ± standard deviation of 3 independent biological replicates. Statistical significance in (D and E) was analyzed using two-way ANOVA with Tukey’s HSD test.

Due to the better outcomes in killing activities of the use of the nucleus-forming jumbophage PhiKZ *in vitro*, we further evaluated the therapeutic efficacy of the phage in *G. mellonella* larvae as an animal infection model. The larvae were injected twice at a 1-h interval with different inoculum combinations as follows: 1) PBS + SM; 2) PAO1 + SM; 3) PBS + either PhiKZ or Callisto (MOI = 100); 4) PAO1 + either PhiKZ or Callisto (MOI = 10); 5) PAO1 + either PhiKZ or Callisto (MOI = 100). The results revealed that all larvae receiving PBS + SM (the negative control) and the phage lysates (PBS + either PhiKZ or Callisto) were able to survive throughout the experiment of 5 days, indicating that the phages were not toxic to the larvae (Figures 5A–5C and S3). The infection of larvae with PAO1 (the positive control) led to very high mortality, leaving only 3% of the survival rate within a 5-day experiment (Figures 5A, 5B, and S3). The treatment by phage Callisto at an MOI of 10, however, did not significantly rescue the infected larvae, resulting in a survival rate of ∼3%, similar to the positive control. Increasing the phage ratio to a MOI of 100 did not much improve the efficacy, resulting in a slight increase in the survival rate to ∼10% (Figures 5A and 5C). Interestingly, the treatment of the infection by the phage PhiKZ had prominent outcomes. The treatment at MOI 10 and MOI 100 substantially decreased the mortality, leading to a survival rate of infected larvae of up to 50% and 60% at day 5, respectively (Figures 5B and 5C).

We also measured the number of viable bacteria and phages in all treatments. Each larva was individually blended, and the number of *P. aeruginosa* and phages was evaluated at the end of the observation period. No viable bacteria were detected in the negative or the phage control (Figure 5D). The results demonstrated that the number of viable bacteria in the phage PhiKZ treatment was 3–4 log lower than that of the phage Callisto treatment (Figure 5D), supporting the high efficiency of therapy with phage PhiKZ over phage Callisto. However, the number of phages at the end of the experiment was comparable in both the phage PhiKZ and Callisto treatments (Figure 5E).

## Discussion

The spatial-temporal organization and immune evasion capabilities exhibited by jumbophages have recently captured the attention of researchers due to their intriguing biological characteristics. Although there has been considerable progress in understanding the fundamental and therapeutic aspects of nucleus-forming PhiKZ-like jumbophages, our understanding of the biological characteristics of T-like jumbophages, particularly their therapeutic potential, remains incomplete. Here, we thoroughly studied the non-nucleus-forming jumbophage Callisto that does not harbor the homolog of ChmA proteins necessary for nuclear formation and then compared it with the well-studied nucleus-forming jumbophage PhiKZ, focusing on two major aspects: biological properties and therapeutic efficacy.

The chimalliviruses PhiKZ and a newly discovered phage Callisto are genetically diverse and speciate into different phage families, resulting in the presence of distinct replication machinery within the bacterial host cells (Figures 2 and 3). Consistent with previous studies,^29^^,^^33^^,^^34^^,^^35^^,^^36^^,^^39^ during infection, the phage PhiKZ assembles the phage nucleus, which is centrally localized at the cell center by the force exerted by the phage spindles. After procapsids are assembled at the cell membrane, they traffic along the filament and dock on the nuclear surface for encapsidation. Subsequently, these DNA-filled capsids detach from the nucleus and localize in the cytoplasm, where they assemble with tails to create mature virions. Unlike the nucleus-forming phage, phage Callisto, which does not harbor the ChmA homolog, is unable to assemble the phage nucleus. Even though the phage does not organize its replication factories similar to the nucleus-forming phages, it tends to arrange its replisomes in a manner similar to the lytic *Bacillus* phage SPP1^56^ and *E. coli* phage λ,^57^ where the replication does not take place within confined boundaries. At late infection, capsids of phage Callisto package the phage genome at several independent locations in the cell where its replisomes are presumably located (Figure S1). This low organization of the phage maturation process in phage Callisto might lead to a reduced number of mature phage virions produced at 60 mpi in comparison to phage PhiKZ, which exhibits a greater level of organization. However, the number of mature virions in both phages appears to be at a comparable level at the end of the replication cycles. Further investigation to explore the mechanisms by which the maturation of phage Callisto is enhanced during the late stage of infection will be needed.

Apart from the different degree of subcellular organization for phage replication, some other reproduction mechanisms are also distinct between these 2 jumbophages. It is also worth noting that, while the adsorption rates between PhiKZ and Callisto are quite similar, the intracellular phage maturation of PhiKZ was six times faster than that of Callisto (Figure S4). This would possibly be a consequence of the compartmentalization during replication that could be beneficial for their multiplication, as suggested by previous studies.^33^^,^^34^^,^^36^^,^^58^^,^^59^ However, it is important to acknowledge that the nucleus-based organization for phage replication alone may not be the exclusive factor responsible for the efficient maturation of PhiKZ progeny. The disparity in the degree of dependence on host replication machinery between the two phages can also contribute to the contrasting effectiveness of phage maturation shown in this study. In particular, Callisto does not possess its own RNAPs, and consequently, the maturation of the phage is heavily dependent on the transcriptional mechanisms of the host, which in turn affects the effectiveness of phage maturation. Further studies are needed in order to dissect whether or how much of the impact of replication compartmentalization, regardless of other factors, plays a role in jumbophage maturation.

As previously mentioned, while phage therapy holds great therapeutic potential, the rapid development of bacterial resistance to phage infection could undermine the therapy’s efficacy.^20^^,^^60^ Based on the presence of the nucleus-like compartment in nucleus-forming phages that functions as a counter-defense mechanism for phages against bacterial immunity, including the DNA-targeting CRISPR-cas system and restriction enzymes,^25^^,^^26^ here we found that PhiKZ, which relies on nucleus-based replication, might offer greater therapeutic potential than non-nucleus-forming jumbophage Callisto, as observed in our *in vitro* and *in vivo* experiments (Figures 4 and 5). In *in vitro* studies, PhiKZ completely suppressed the bacterial growth over a duration of 18 h at all ranges of MOIs, even at MOIs as low as 0.01. As observed in Callisto, which displays lower subcellular organization and does not assemble the protective shield for its genome, the bactericidal activity of Callisto is not as potent as that of phage PhiKZ. Even though Callisto was used at a high MOI (100), the emergence of phage resistance occurred rapidly within 12 h of treatment. These findings may be attributed to the existence of the nucleus-like structure, which circumvents bacterial defense mechanisms^25^^,^^26^^,^^61^ and consequently mediates the high treatment efficiency observed in the phage PhiKZ treatment. However, some viable bacteria that survived through the phage PhiKZ treatment were detected after 24 h. This might be a result of the bacterial defense via RNA-targeting immunity that can degrade the viral mRNAs upon their export into the cytoplasm for protein translation.^62^ Since the anti-phage system is a dynamic evolutionary process that involves an ongoing arms race between phages and their bacterial hosts and is definitely not limited to the exclusion of bacterial defense through nucleus formation, further investigation into other mechanisms employed by hosts to overcome phage invasions will be required. In corresponding with the *in vitro* studies, the *in vivo* studies in *G. mellonella* larvae (greater wax moth)^63^ also reveal that the nucleus-forming jumbophage PhiKZ exhibits stronger therapeutic efficacy than phage Callisto. This is evidenced by a significant reduction of the bacterial load in the infected animals up to 3–4 logs, thus enhancing a 6-fold higher survival rate compared to the use of Callisto. The titers of both phages in all infected animals at the end of the experiment, however, are all at a comparable level (Figure 5E), indicating no correlation between the effectiveness of the therapy and the phage quantity. Further comprehensive research that considers several nucleus-forming and non-nucleus-forming jumbophages is truly necessary to determine if the organized replication machinery plays significant roles in superior antibacterial activity, reducing the emergence of phage resistance, and improving therapeutic outcomes.

While the biological features of nucleus-forming jumbophages may offer certain advantages over non-nucleus-forming jumbophages, it is not a foolproof criterion for selecting potential therapeutic phage candidates. The combination of a diverse range of phages with different characteristics (i.e., receptor preference or replication machinery) in a phage cocktail instead contributes to enhancing the overall efficacy of phage therapy.^64^ For example, utilizing genetically diverse phages that exhibit distinct mechanisms of pre-killing (MOK) is shown to be a superior approach compared to employing closely related phages or single phage alone.^41^ In particular, our jumbophages Callisto and PhiKZ, which are proven here to display different MOKs, would possibly be potential candidates for an effective phage combination for further investigation of their use in clinical settings. It is worth noting that some jumbophages might have drawbacks for application due to their nature: a long latent period and small burst size,^27^^,^^28^ and these factors should definitely be taken into account when selecting jumbophage candidates for therapy. Moreover, phages have a wide range of applications, and their use is not restricted to the combination of themselves. Given the unavoidable prevalence of phage-resistant mutants, numerous studies have focused on utilizing combinations of phages and antibiotics, with a particular interest in their synergistic effects for therapeutic applications.^65^^,^^66^ This is intriguing because the effectiveness of phage-antibiotic therapy is shown to rely on multiple parameters, especially the phage characteristics.^67^^,^^68^^,^^69^ Therefore, we believe that an in-depth understanding of phage characteristics will serve as a critical step in selecting a potent candidate for combination therapy (phage-phage and phage-antibiotic) with favorable outcomes for future jumbophage-based therapeutic applications.

### Limitations of the study

This current study demonstrates the relationship between the degree of subcellular organization and the therapeutic efficiency of jumbophages. However, the direct impact of replication compartmentalization, regardless of other factors, on therapeutic outcomes needs further investigation. Indeed, more nucleus-forming and non-nucleus-forming jumbophages will be required to strengthen the finding of this study.

## STAR★Methods

### Key resources table

**Table:** 

REAGENT or RESOURCE	SOURCE	IDENTIFIER
**Bacterial and virus strains**		

*P. aeruginosa* PAO1	Pogliano strain collection	N/A
*P. aeruginosa* PSU-PA01	Surachat Lab	N/A
*P. aeruginosa* PSU-PA02	Surachat Lab	N/A
*P. aeruginosa* PSU-PA03	Surachat Lab	N/A
*P. aeruginosa* PSU-PA04	Surachat Lab	N/A
*P. aeruginosa* PSU-PA05	Surachat Lab	N/A
*P. aeruginosa* PSU-PA06	Surachat Lab	N/A
*P. aeruginosa* PSU-PA07	Surachat Lab	N/A
*P. aeruginosa* PSU-PA08	Surachat Lab	N/A
*P. aeruginosa* PSU-PA09	Surachat Lab	N/A
*P. aeruginosa* PSU-PA10	Surachat Lab	N/A
*P. aeruginosa* PSU-PA11	Surachat Lab	N/A
*P. aeruginosa* PSU-PA13	Surachat Lab	N/A
*P. aeruginosa* PSU-PA14	Surachat Lab	N/A
*P. aeruginosa* ATCC9027	American Type Culture Collection	N/A
*P. aeruginosa* ATCC15442	American Type Culture Collection	N/A
*P. aeruginosa* ATCC27853	American Type Culture Collection	N/A
*P. stutzeri* DMST28410	Department of Medical Sciences Thailand	N/A
*P. stutzeri* DMST12562	Department of Medical Sciences Thailand	N/A
*P. mendocina* ATCC25411	American Type Culture Collection	N/A
*P. fluorescens* ATCC13525	American Type Culture Collection	N/A
*P. putida* ATCC12633	American Type Culture Collection	N/A
*P. putida* ATCC17522	American Type Culture Collection	N/A
NEB 5-alpha Competent *E. coli*	New England Biolabs	Cat#C2987H
*P. aeruginosa* K2733	Keith Poole of Queen’s University Kingston, ON, Canada	N/A
*P. aeruginosa* phage PhiKZ	https://doi.org/10.1006/jmbi.2001.5396	N/A
*P. aeruginosa* phage Callisto	Chaikeeratisak strain collection	N/A

**Chemicals, peptides, and recombinant proteins**		

Tryptone Type-1	HIMEDIA	CAS: RM014-500G
NaCl	KEMAUS	CAS: 7647-14-5
Yeast extract powder	HIMEDIA	CAS: RM027-500G
Agar powder, Bacteriological grade	HIMEDIA	CAS: GRM026-500G
MgSO4·7H2O	EMSURE® ACS	CAS: 10034-99-8
Tris	ACS,Reag. Ph Eur	CAS: 77-86-1
Uranyl acetate	Electron Microscopy Sciences	CAS: 22400-1
DNaseI	Thermo Scientific™	CAS: EN0525
RNaseA	Thermo Scientific™	CAS: R1253
proteinase K	Invitrogen™	CAS: AM2542
EDTA	LOBA CHEMIE PVT.LTD.	CAS: 6381-92-6
SDS	SIGMA	CAS: 75746-250G
phenol: chloroform: isoamyl alcohol (25:24:1)	SIGMA	CAS: 77617-100ML
Chloroform	SIGMA	CAS: C2432-500ML
FM 4–64	Invitrogen™	CAS: T13320
DAPI	Invitrogen™	CAS: D1306
Gentamycin sulfate	Sigma-Aldrich	Cat#G1914
Na2HPO4	QReC	CAS: 7758-79-4
Rifampicin	TCI	CAS: 13292-46-1
Polyethylene glycol	LOBA CHEMIE PVT.LTD.	CAS: 25322-68-3

**Critical commercial assays**		

Phusion High-Fidelity DNA Polymerase	New England Biolabs	Cat#M0530L
Deoxynucleotide (dNTP) Solution Mix	New England Biolabs	Cat#N0447L
MacConkey agar	Difco, BBL™	CAS: 90004-036
NEBuilder HiFi DNA Assembly Cloning Kit	New England Biolabs	Cat#E5520S
Presto™ Mini Plasmid Kit	Geneaid	Cat. # PD100
PCR Cleanup Kit	Geneaid	Cat. # DFC100

**Deposited data**		

*P. aeruginosa* phage Callisto	This paper, NCBI	GenBank: OR142618

**Experimental models: Organisms/strains**		

Caterpillars, *Galleria mellonella*	Local vendor, Thailand	N/A

**Oligonucleotides**		

See supplemental materials	This manuscript	Table S3

**Recombinant DNA**		

pHERD30T plasmid	Dr. Hongwei D Yu of Marshall University	N/A
gp48-GFPmut1/pHERD30T	This study	pAN036
gp51-GFPmut1/pHERD30T	This study	pAN053
gp292-GFPmut1/pHERD30T	This study	pAN062
gp300-GFPmut1/pHERD30T	This study	pAN064
gp394-GFPmut1/pHERD30T	This study	pAN066
gp154-GFPmut1/pHERD30T	This study	pAN068

**Software and algorithms**		

Spades 3.11.1	Bankevich et al.^70^	https://doi.org/10.1089/cmb.2012.0021
PHASTER	Arndt et al.^71^	https://phaster.ca/
GeneMarkS	Lukashin et al.^72^	https://doi.org/10.1093/nar/26.4.1107
VIRIDIC	Moraru et al.^73^	https://rhea.icbm.uni-oldenburg.de/VIRIDIC/
Easyfig	Sullivan et al.^74^	http://mjsull.github.io/Easyfig/
VICTOR web	Meier-Kolthoff et al.^75^	https://ggdc.dsmz.de/victor.php
iTOL	Letunic et al.^76^	https://itol.embl.de/login.cgi
FIJI/ImageJ	NIH	https://imagej.nih.gov/ij/
Adobe Photoshop	Adobe	https://www.adobe.com/products/photoshop.html
Adobe Illustrator	Adobe	https://www.adobe.com/products/illustrator.html
Microsoft Excel	Microsoft Office	https://products.office.com/en-us/excel
SPSS version 29.0.1	SPSS, Inc., Chicago, IL, USA	Concurrent user license
OriginPro 2023	OriginLab	https://www.originlab.com/2023
Cellprofiler 4.0 software	McQuin et al.^77^	https://doi.org/10.1371/journal.pbio.2005970
CGView		http://stothard.afns.ualberta.ca/cgview_server/
tRNAscan-SE	Chan et al.^78^	tRNAscan-SE Search Server (ucsc.edu)

### Resource availability

#### Lead contact

Further information and requests for resources and reagents should be directed to and will be fulfilled by the lead contact, Vorrapon Chaikeeratisak (vorrapon.c@chula.ac.th).

#### Materials availability

‘‘This study did not generate new unique materials.’’

#### Data and code availability

• All data reported in this paper will be shared by the lead contact upon request.
• This paper does not report original code.
• Any additional information required to reanalyze the data reported in this paper is available from the lead contact upon request.

### Experimental model and subject details

#### Animals

All animal husbandry and procedures were performed in accordance with institutional guidelines as approved by the Certification of Institutional Animal Care and Use Committee (IACUC) in accordance with university regulations and policies governing the care and use of laboratory animals under protocol number 2423001. The review has followed guidelines documented in Ethical Principles and Guidelines for the Use of Animals for Scientific Purposes edited by the National Research Council of Thailand. All experiments were undertaken using approximately 25-35-day-old caterpillars purchased from Local vendor, Thailand. A total of 30 caterpillars were placed in plastic containers (+/− 30 cm high) with holes in the covers and maintained under dark containers or protect the containers from light at 28°C.

### Method details

#### Bacterial strains and growth conditions

Twenty-three *Pseudomonas* strains were used in this study including 17 *P aeruginosa* strains, 2 *P stutzeri* strains, 2 *P putida* strains, *P. mendocina* ATCC25411, and *P. fluorescens* ATCC13525. Clinal *P. aeruginosa* strains PSU-PA01–PSU-PA14 were isolated from patients in internal medicine at Songklanagarind Hospital, Thailand (See also STAR Methods). The bacteria were cultured on LB broth (10 g/L tryptone, 10 g/L NaCl, and 5 g/L yeast extract) at 37°C, followed by shaking at 250 rpm. When plated, cells were grown on LB containing 1.5% agar and incubated inverted at 37°C.

This work has been reviewed and approved by Chulalongkorn University-Institutional Biosafety Committee (CU-IBC) in accordance with the levels of risk in pathogens and animal toxins, listed in the Risk Group of Pathogen and Animal Toxin (2017) published by the Department of Medical Sciences (Ministry of Public Health), the Pathogen and Animal Toxin Act (2015) and Biosafety Guidelines for Modern Biotechnology BIOTEC (2016) with approval number: SC CU-IBC-028/2020 Ex1.

#### Phage isolation and its biological characteristics

##### Phage isolation

Water samples collected from a reservoir in Chulalongkorn University, Bangkok, Thailand (coordinates on a map: 13.73804, 100.53183) were mixed with *P. aeruginosa* PAO1 and incubated overnight prior to phage enrichment. Briefly, water samples were filtered through a membrane filter, 0.45 μm pore size. The filtrates were mixed with 2X LB medium and log phase culture of PAO1 was then added, followed by incubation at 37°C with shanking at 80 rpm for 24 h. After centrifugation, the supernatant was mixed with LB broth containing 0.5% agar powder and subsequently combined with the host. The mixture was overlaid onto an LB plate and incubated at 37°C for 24 h to observe plaque formation. The isolated plaque was collected and re-plated five times to ensure single phage purification. The purified phage was designated as phage Callisto. Phage stocks were maintained in SM buffer with gelatin (100 mM NaCl, 8 mM MgSO_4_·7H_2_O, 50 mM Tris-Cl, pH 7.5 caution 0.01% (w/v) gelatin) and subsequently stored at 4°C.

##### Phage propagation

Twenty milliliters of the parental host culture (1 × 10^6^ CFU/mL) were infected at a multiplicity of infection (MOI) of 0.1 followed by incubation at 37°C with shaking at 80 rpm for 24 h. The host cell was removed by centrifugation. The supernatant was filtrated through a membrane filter, 0.45 μm pore size, and subsequently stored at 4°C for further experiments. To determine phage concentration, a 10-fold serial of phage stock was spotted on the lawn of the parental host and incubated overnight at 37°C. The number of phages was calculated by plaque observation and expressed as plaque-forming units (PFU) per mL.

##### Host range

The ability of phages against different 23 *Pseudomonas* strains was performed. Five microliters of phage were dropped onto the lawn of each host. The plate was incubated at 37°C for 24 h to observe the lytic zone. The experiment was undertaken in triplicate. The efficiency of plating (EOP) value is calculated as the ratio of the PFU value of phage with susceptible bacterial strain and the phage with the parental host.

##### Effects of temperature, and pH on phage viability

The stability of phages at different conditions in SM buffer was investigated. The various condition of temperatures (25°C, 30°C, 37°C, 40°C, 50°C, 60°C, and 70°C) and pHs (2, 4, 6, 8, 10, and 12) were set. The phage stock was diluted to a final concentration of 10^6^ PFU/mL in 1 mL of SM buffer and incubated in the indicated conditions for 6 h. Subsequently, phage titers were measured as described above. The experiments were conducted in triplicate.

##### Phage morphology under transmission electron microscopy (TEM)

TEM was undertaken to visualize phage morphology by the negative staining method. Ten microliters of phage stock were spotted in the lawn of the parental host and incubated overnight at 37°C. A fresh clear zone was collected and suspended in the SM buffer without gelatin followed by incubation at 4°C overnight. The supernatant was filtrated through a membrane filter, 0.45 μm pore size, placed on copper grids, and negatively stained with 2% (w/v) uranyl acetate (pH 4.5). Phage morphology was observed with a Hitachi HT7700 transmission electron microscope at 80 kV.

#### Phage growth characteristics

##### Phage adsorption

The phage adsorption curve was determined as follow: The host cells were grown to log phase, then infected with phages at an MOI of 0.1 followed by incubation at 37°C. Aliquots of 300 μL were taken at an interval of every 10 min for 60 min and immediately filtrated through a membrane filter, 0.45 μm pore size. Independent experiments were performed in triplicate.

##### One-step growth curve

One-step phage growth curve was performed. Five milliliters of log phase host culture were centrifuged and resuspended in 1 mL of LB. PAO1 was infected with phage at an MOI of 0.1 and incubated at 37°C for 20 min for phage adsorption. The pellet containing infected cells was harvested, resuspended in 50 mL of LB broth, and then incubated at 37°C. Samples were taken at 10 min intervals for 170 min and subsequently filtrated through 0.45 μm. The titer of phages was immediately determined using the double-layer technique. Briefly, 100 μL of filtrate and 200 μL the parental host were pipetted on LB plate and mixed with 5 mL of soft LB agar immediately, followed by incubation at 37°C overnight. Independent experiments were undertaken in triplicate. The latent period and burst size were estimated as described elsewhere.^79^ The burst size was calculated: (Average phage number at the plateau phase - Average phage number at the latent phase)/The initial phage yield.

#### Phage genome sequencing and analysis

The phage DNA was extracted using a previous report with modification.^80^ Briefly, purified phage was treated with DNaseI (2 mg/mL) and RNaseA (100 mg/mL) overnight at 37°C to remove contaminated host DNA and RNA. Subsequently, the lysis buffer (1M Tris, pH 8.0, 0.5M EDTA, pH 8.0, 10% SDS, and 10 mg/mL proteinase K) was added followed by incubation at 60°C for 1 h. The phage DNA was extracted twice with phenol: chloroform: isoamyl alcohol (25:24:1) and chloroform. After centrifugation, the supernatant containing DNA was collected and precipitated by mixing 0.3 volume of 3 M NaOA and 1 volume of isopropanol followed by incubation for 2 h at −20°C. After that, the pellet was washed with 70% ethanol followed by centrifugation. The pellet was air-dried, dissolved in sterile distilled water, and then kept at −20°C. The DNA quality was determined by measuring the 260/280 ratio and then the DNA concentration was estimated by a nanodrop.

The whole-genome sequencing of phage DNA was performed using short-read sequencing through MGISEQ-2000 (Beijing Genomics Institute, Beijing, China). The quality of reads was checked with FASTQC and filtered using Trimmomatic 0.39 with default parameters. Spades 3.11.1 was used to assemble all reads into contigs. The open reading frames (ORFs) were identified using PHASTER and GeneMarkS followed by annotation with Blastp of the NCBI server. The tRNA was investigated using tRNAscan-SE. Intergenomic distances/similarities among viral genomes were determined by the VIRIDIC (Virus Intergenomic Distance Calculator) web-service. The genome map was conducted by CGView. A linear comparison of multiple genomic loci was investigated by Easyfig. Phylogenetic trees were constructed using the Genome-BLAST Distance Phylogeny (GBDP) method and generated with the VICTOR web service with 100 bootstraps and visualized with iTOL. The phage genome was deposited in NCBI (OR142618; https://www.ncbi.nlm.nih.gov/nuccore/OR142618).

#### Single-cell infection level and image data analysis

##### Single-cell assay

PAO1 was cultured to reach OD_600_ ∼ 0.4 and infected with phages at a MOI of 10 followed by incubation at 37°C for 30, 60, and 90 min. The infected cells were fixed at a final concentration of 4% paraformaldehyde and incubated at room temperature for 15 min as described by Chaikeeratisak et al.*.*^34^ The fixed cells were harvested by centrifugation and then washed with 1 mL of 1x PBS twice. The cells were resuspended in 1x PBS and strained with fluorescent dyes (2 μg/mL FM 4–64 and 2 μg/mL DAPI). After centrifugation, 3 μL of cell suspension was loaded onto an agarose pad (1.2% agarose in 20% LB). The samples were visualized under Delta Vision Ultra High-Resolution Microscope.

##### Qualitative phage infection under transmission electron microscopy (TEM)

To determine the intracellular phage-like particles, PAO1 was infected with phage at a MOI of 10 and PAO1 cultures without phage inoculation served as a control. At 60 min post inoculation, both PAO1 cultures were fixed with 2% glutaraldehyde in phosphate-buffered saline (13 mM NaH_2_PO_4_·2H_2_O, 86.8 mM Na_2_HPO_4_·12H_2_O, 85.6 mM NaCl; pH 7.4) and stored at 4°C. Post-fixation was mixed with 1% osmium tetroxide in PBS buffer, dehydrated in a graded ethanol series (50–100%), infiltrated with acetone, and embedded in Epon resin. Ultrathin sections (90–100 nm) were stained with 1% uranyl acetate and 3% lead citrate and observed at 80 kV using a Hitachi HT7700 transmission electron microscope.

##### The cytological profile

Raw images from the fluorescence microscope were pre-processed on ImageJ software. Then, all individual cells were extracted for morphological features by automatic cell analysis using Cellprofiler 4.0 software.^77^ The morphological feature set was selected based on the previous study.^81^^,^^82^ Statistical calculation and machine learning are carried out using the scikit-learn library in python.^83^ Briefly, cell profile data were transformed and normalized with Cube Root Transformation and Z-scored normalization,^84^ respectively. Finally, the dimension of the dataset was reduced and visualized with Uniform Manifold Approximation and Projection (UMAP).^85^ To compare the uninfected and infected cell profiles, the uninfected cell of the phage treatment group was excluded by selecting the infected cell cluster on the UMAP plot that does not overlap with the uninfected cell cluster.

##### Plasmids construction and fluorescence microscopy

Genes related to replisomes (gp48, gp51, and gp394), DNA packaging (gp154) and virion structure (gp292 and gp300) were studied (Table S2). All genes were amplified from phage DNA using polymerase chain reaction amplification using specific primers (Table S3) followed by the insertion of each amplicon into the linearized pHERD30T containing GFPmut1 fluorescent proteins using the NEBuilder HiFi DNA Assembly Cloning Kit to obtain a construct (key resources table and Table S2). The resulting recombinant plasmids were transformed into *Escherichia coli* DH5ɑ by the electroporation method and then grown in LB plates supplemented with 15 μg/mL gentamicin at 30°C. The correctness of the recombinant plasmid was confirmed by DNA sequencing, and they were later electroporated into *P. aeruginosa* K2733 and grown in an LB plate supplemented with 5 μg/mL gentamicin at 30°C to create strains, as listed in Table S2.

To visualize the infected cells containing the indicated construct, overnight cultures of *P. aeruginosa* K2733 grown at 37°C were diluted 1:100 in fresh LB with arabinose at the indicated concentration, and grown at 37°C. At an OD_600_ of 0.4 the cultures were infected with the Callisto at a MOI of 10 followed by incubation for 20 min. Infected cells were transferred on agarose pads containing 1 μg/mL FM4-64 for membrane staining and 2 μg/mL DAPI for DNA staining and microscopy was carried out subsequently at 37°C. Image acquisitions were performed on a Zeiss Axio Observer.

##### Quantitation of intracellular infection

The number of infectious particles was quantified by a previous report with modification.^56^ Log phase PAO1 cultures infected with phage at a MOI of 10 at 37°C were collected at 60 and 90 min. After centrifugation, the infected cells were washed twice with PBS. To release phage particles in bacteria, the infected cells were lysed with 10% chloroform at the final concentration followed by incubation on ice for 1 h. Then, the mixture was centrifuged and the phage in the aqueous phase was tittered to determine the intracellular phages per bacterial cell. Each independent trial was repeated three times.

##### Effect of rifampicin on phage propagation

To investigate whether Callisto replication depends on the host transcriptional machinery, rifampicin was used as an inhibitor for host RNA synthesis. Since the PhiKZ replication in PAO1 was shown to be resistant to rifampicin treatment,^86^ it is used as the control experiment. The log phase of PAO1 culture (at OD_600_ ∼ 0.4) were added with rifampicin (0, 100, or 400 μg/mL at the final concentration) and incubated for 30 min. The cultures were infected with phages at a MOI of 10 and incubated for 60 min at 37°C with shaking at 80 rpm. The number of intracellular phage particles was determined at 90 mpi as described above. The experiment was performed in independent triplicate.

#### The bactericidal activity of the phages *in vitro*

To evaluate phage lytic ability, killing curves were determined on PAO1 in the presence of phages at an MOI of 0.01, 0.1, 1, 10, and 100. Briefly, the log-phase bacterial cells were loaded into a 96-well microplate and mixed with the indicated MOIs of phage in 200 μL LB medium. The optical density of the plate was measured automatically every 10 min interval up to 240 min at a wavelength of 600 nm. To verify the antibacterial efficacy of phage after infection at an MOI of 1, 10, or 100, bacterial number and phage number were counted at the indicated sampling time (24 and 48 h). Each independent trial was repeated three times.

#### The bactericidal activity of the phages *in vivo*

To evaluate the therapeutic efficacy of PhiKZ and Callisto against PAO1, a *Galleria mellonella* larva infection was used as described previously. The larvae were obtained from a local vendor and stored in the dark for 10 days. Healthy larvae weighing ranging from 200 to 250 mg (10 larvae) were selected randomly and swabbed with 70% ethanol followed by keeping in a 90-mm Petri dish in darkness overnight prior to the test. The PAO1 culture at log phage was centrifuged, and the pellet was washed twice with PBS. The larvae were divided into the following 8 groups of 10 larvae each: (1) control, PBS+SM; (2) bacterial infection, PAO1 (1 × 10^5^ CFU/mL) +SM; (3) PhiKZ phage-only, 1 × 10^7^ PFU/mL+PBS; (4), Callisto phage-only, 1 × 10^7^ PFU/mL+PBS; (5) phage treatment at an MOI of 10, PAO1 (1 × 10^5^ CFU/mL) + PhiKZ (1 × 10^6^ PFU/mL); (6) phage treatment at an MOI of 100, PAO1 (1 × 10^5^ CFU/mL) + PhiKZ (1 × 10^7^ PFU/mL); (7) phage treatment at an MOI of 10, PAO1 (1 × 10^5^ CFU/mL) + Callisto (1 × 10^6^ PFU/mL); (8) phage treatment at an MOI of 100, PAO1 (1 × 10^5^ CFU/mL) + Callisto (1 × 10^7^ PFU/mL). Ten microliters of PAO1 were injected into the last right-side prolonged leg of the larvae using an insulin syringe (Ultra-Fine II), and then incubated in the dark in 90-mm plastic petri dishes for 1 h. The phages at 10 μL at the indicated concentration were injected into the last left-side prolonged leg. The mortality of the larvae was observed daily for 5 days, and the Kaplan-Meier survival curves were plotted. At 5 days, bacterial and phage numbers were determined by culturing on MacConkey agar and on a double layer agar plaque assay, respectively. Each independent trial was repeated three times.

### Quantification and statistical analysis

All graphs were visualized in OriginPro 2023. Experiments were subject to ANOVA with SPSS version 28.0.1 (SPSS, Inc., Chicago, IL, USA) described in the corresponding figure legends.
