# Supplementary material for: Nucleus-forming jumbophage PhiKZ therapeutically outcompetes non-nucleus-forming jumbophage Callisto
Source: iScience. 2024 Apr 18;27(5):109790. doi: 10.1016/j.isci.2024.109790 (PMC11079468; doi:10.1016/j.isci.2024.109790)
Supplement: Document S1. Figures S1–S4 and Tables S1–S3 [file mmc1.pdf]

iScience, Volume *27*

Supplemental information

Nucleus-forming jumbophage PhiKZ therapeutically outcompetes non-nucleus-forming jumbophage Callisto

Ampapan Naknaen, Thanadon Samernate, Panida Saeju, Poochit Nonejuie, and Vorrapon Chaikeeratisak

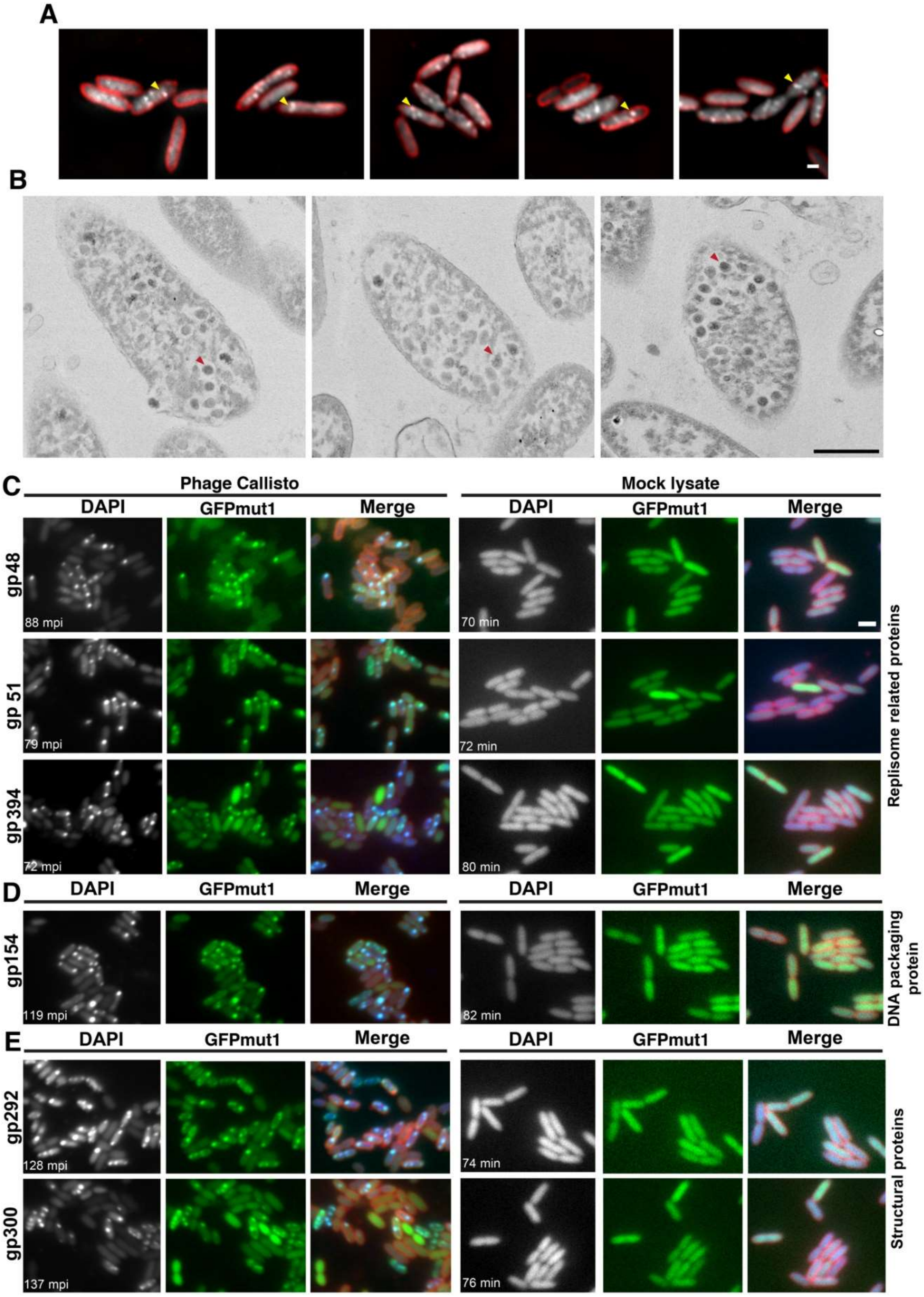

Figure S1. Microscopic images of phage Callisto-infected *P. aeruginosa* and its replisomes, related to Figure 3.

(A) Still fluorescent images (scale bar equals 1 micron), **(B)** Conventional ultrathin sectioning and transmission electron microscopy images of *P. aeruginosa* infected with phage Callisto at 60 mpi (scale bar equals 500 nm) and **(C-E)** The DNA packaging of phage Callisto occurs in multiple viral replisomes during the infection (the scale bar equals 2 μm).

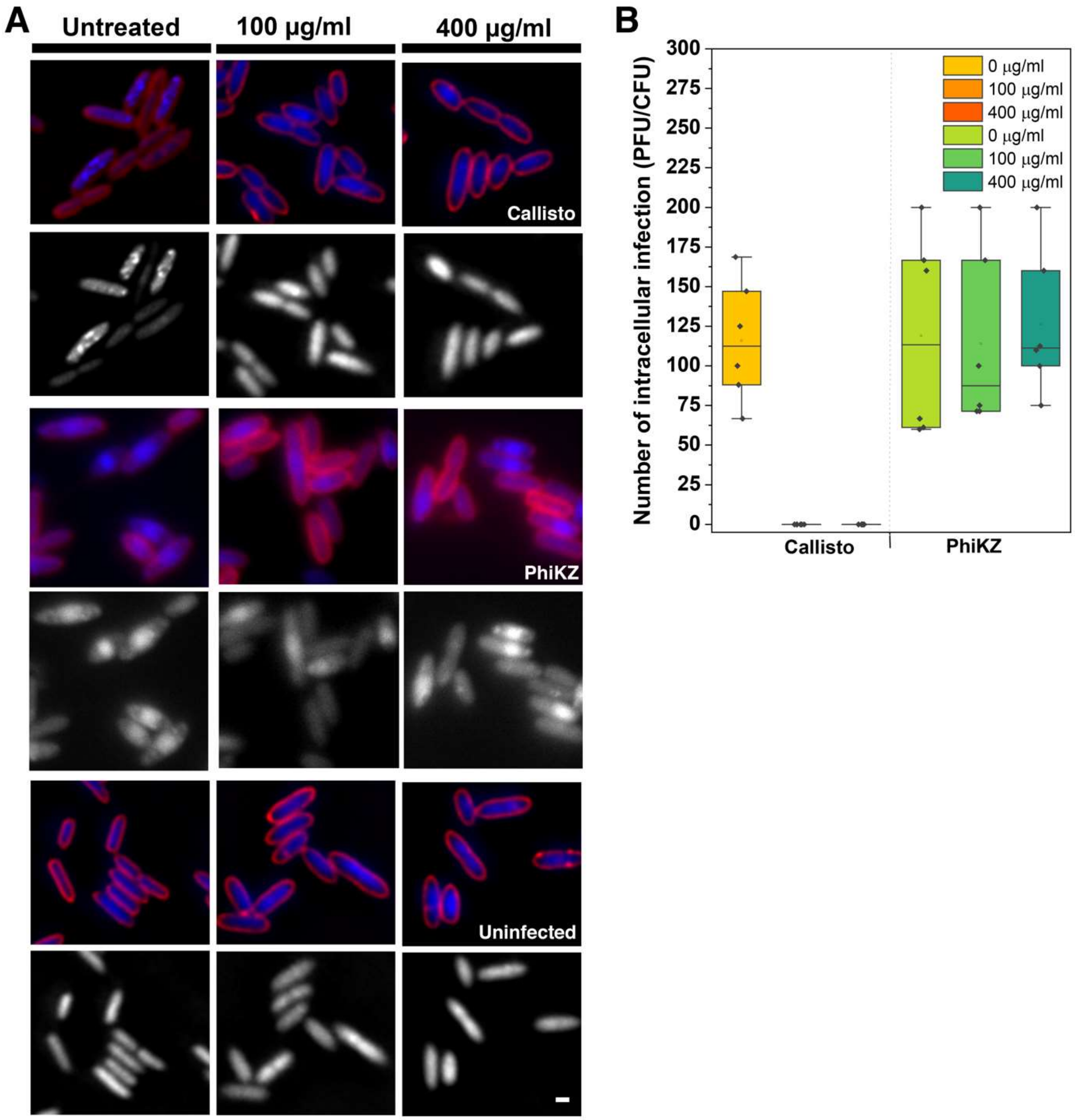

Figure S2. Jumbophage Callisto replication relies on host RNA transcription machinery, related to Result and Figure 3.

(A) *P. aeruginosa* infected with either Callisto or PhiKZ at 60 mpi upon the treatment with 100 µg/ml and 400 µg/ml rifampicin (Scale bar equals 1 micron) and **(B)** the number of intracellular phage particles (PFU/CFU) of Callisto and PhiKZ at 90 mpi after rifampicin treatment.

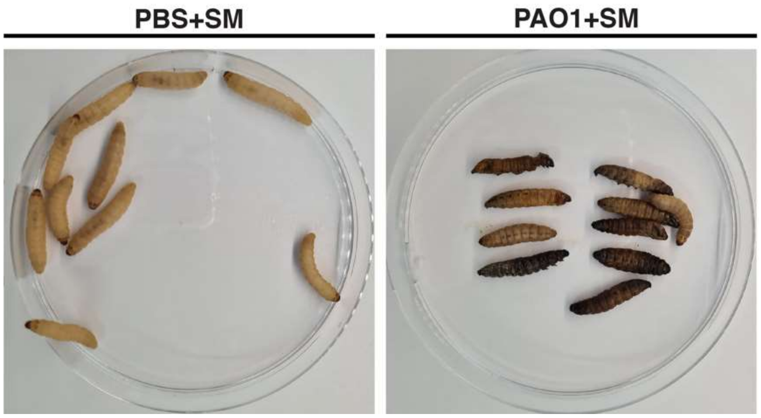

Figure S3. The control experiments of *in vivo* study, related to Figure 5.

Larvae injected with PBS+SM (the negative control) showed movement without melanization while larvae injected with PAO1+SM (the positive control) appeared black (melanization) with no movement within 1 day.

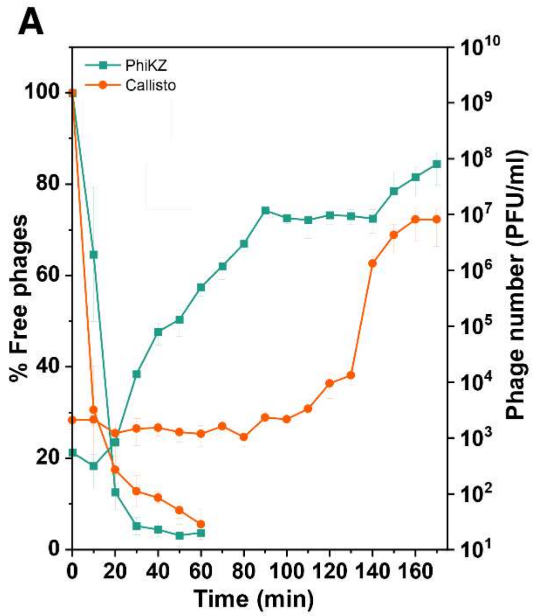

B

Phage growth parameters	Callisto	PhiKZ
Ka (ml/min)	2.56×10^{-9}	3.37×10^{-9}
Eclipse period (E;min)	80	20
Latent period (L;min)	110	30
Burst size (B, phage/cell)	70 ±25	123 ±57
Intracellular rate of phage progeny maturation (phages/min)	2 ±0.9	12 ±6.0

Figure S4. Comparison of phage infection and growth characteristics of phage Callisto and PhiKZ, related to Discussion and Figure 4.

(A) Adsorption rate and one-step growth curve of non-nucleus-forming phage Callisto (orange) and nucleus-forming phage PhiKZ (green) using PAO1 as the host in LB medium at MOI of 0.1. **(B)** Growth parameters derived from the kinetic growth curve; the *ka* values (ml/min) were determined by analysis of the adsorption curves; the rate of progeny maturation (phages/min) could be measured as the ratio of the phage burst size (B; burst size) to the duration of the phage period of progeny maturation (L; latent period, subtracts from E; eclipse period).

Table S1. The functional annotation of ORFs in phage Callisto, related to Result and Figure 1.

	ORFs	CDS Position	BLAST Hit	Accession	E-value
1	1	49..1872	fusion long tail fiber distal subunit	NC_021529	3.62E-17
2	3	complement(2480..2797)	MutT-like protein	NC_025447	4.27E-31
3	7	complement(5946..7481)	tail fiber repeat family protein	NC_023863	5.00E-07
4	8	complement(7483..9081)	putative DNA methylase	NC_042074	9.19E-10
5	14	complement(13141..13656)	terminase large subunit	NC_041880	3.33E-39
6	17	14422..14892	putative transcriptional regulator	NC_042133	3.18E-26
7	18	14889..15134	putative permease	NC_021529	1.73E-10
8	22	16602..16853	glutaredoxin	NC_020201	1.77E-12
9	27	18524..20707	putative rIIA protector from prophage-induced early lysis	NC_028773	1.49E-57
10	28	20700..21443	protector from prophage-induced early lysis	NC_023561	5.61E-59
11	29	21527..23548	Oligopeptidase A	YP_010678918.1	0.00E+00
12	36	27182..29821	DNA primase	NC_042013	1.22E-160
13	37	29923..31539	major tail protein	NC_031039	1.18E-56
14	38	31628..32248	recombination endonuclease subunit D12	NC_042013	3.24E-50
15	41	34241..34669	head completion protein	NC_019526	3.59E-35
16	42	34671..35177	ATP-dependent helicase	NC_042013	1.11E-06
17	45	37368..38963	L shaped tail fiber protein	NC_041917	2.27E-153
18	48	40514..42004	single strand DNA binding protein	WPK39713.1	0.00E+00
19	49	43296..43541	side tail fiber	NC_042116	6.09E-18
20	51	43594..45027	RecA-like proteins	UYE98703.1	2.00E-124
21	52	46295..48130	terminase large subunit	NC_043027	4.55E-53
22	55	48627..49091	distal tail protein	NC_041878	1.80E-33
23	56	49104..50555	distal tail protein	NC_041917	8.69E-155
24	59	51322..51789	single-stranded-DNA-specific exonuclease	NC_042013	1.96E-13
25	60	51803..52510	tape measure chaperone	NC_041917	1.52E-37
26	64	54464..55486	terminase small subunit	NC_042013	4.18E-50
27	65	55488..56927	DNA primase-helicase subunit	NC_027399	5.20E-79
28	81	66813..67478	tail completion protein	NC_042116	9.95E-55
29	86	72672..73694	baseplate hub subunit and tail lysozyme	NC_025422	1.53E-29
30	92	75665..76561	DNA adenine methyltransferase	NC_042140	1.32E-60
31	94	77021..77710	GTP cyclohydrolase I	NC_005083	8.80E-86
32	99	80073..80543	QueD-like queosine biosynthesis protein	NC_048804	6.11E-29
33	100	80627..81058	heat shock protein	NC_015282	9.87E-20
34	101	81173..81475	ATP-dependent Clp protease adaptor protein	NC_042013	8.26E-09
35	102	81550..82098	Superoxide dismutase [Mn/Fe]	NDC22864.1	1.00E-129
36	107	83731..85461	methyltransferase type 11	NC_028998	1.71E-16
37	108	85489..86859	DNA ligase	VOH54155.1	0.00E+00
38	111	87656..88429	DNA polymerase III subunit epsilon	BDR25777.1	0.00E+00
39	113	88920..89591	head maturation protease	YP_010679002.1	6.00E-165
40	119	91789..92268	tRNA-specific adenosine deaminase	VOH54238.1	4.00E-114
41	113	97294..98223	Holliday junction ATP-dependent DNA helicase RuvB	CAB4130327.1	2.00E-134

42	136	complement(100029..99256)	PhoH-like protein	YP_010679024.1	0.00E+00
43	139	101497..102186	Chaperone protein DnaJ	YP_010679027.1	5.00E-164
44	140	102196..104415	ATP-dependent Clp protease ATP-binding subunit ClpA	BDR26166.1	0.00E+00
45	150	121902..122849	N-acetylneuraminate epimerase	VOH54459.1	0.00E+00
46	154	127576..129258	HNH homing endonuclease III	VOH54489.1	0.00E+00
47	159	133184..134245	ATP-dependent helicase;	NC_041997	1.42E-55
48	166	137311..137922	clamp loader, small subunit	NC_042013	4.36E-23
49	167	137922..138908	RNaseH ribonuclease	NC_019401	3.65E-99
50	168	138911..139369	deoxi-UTP pyrophosphatase	NC_042013	2.45E-09
51	172	140499..140792	resolvase	NC_042013	2.93E-31
52	256	183597..183827	DksA/TraR family C4-type zinc finger protein	YP_010679141.1	1.00E-47
53	264	185910..186521	tRNA(Ile)-lysidine synthase	YP_010679150.1	4.00E-150
54	292	196399..196716	Structural protein	YP_010678863.1	1.00E-25
55	300	200071..200229	Structural protein	YP_010678736.1	1.00E-55
56	394	261323..261607	putative DNA polymerase	YP_010678863.1	6.00E-61

Table S2. Callisto proteins including replisome-related proteins, DNA packaging proteins, and structural proteins studied in this work, related to STAR Methods.

Callisto proteins	Strain number	Plasmid name	Host
**Replisome proteins**			
gp48 (single-stranded DNA binding protein)	AN_1036	pAN036	*K2733*
gp51 (RecA-like protein)	AN_1083	pAN053	*K2733*
gp394 (putative DNA polymerase)	AN_1096	pAN066	*K2733*
**DNA packaging protein**			
gp154 (HNH homing endonuclease III)	AN_1098	pAN068	*K2733*
**Virion Structural proteins***			
gp292	AN_1092	pAN062	*K2733*
gp300	AN_1094	pAN064	*K2733*

*These proteins were identified as structural proteins of PA5oct phage using ESI-MS/MS[48].

Table S3. Oligonucleotides used in this study, related to STAR Methods.

**Callisto proteins**	**Forward Primer (5'-3')**	**Reverse Primer (5'-3')**	**Size of genomic fragment deleted (bp)**
gp48	AGGAGATATACATACCC GGC<br>ATGGCTTCTCTTGCTGA	GCAGCGGCCGCTCCGGAGGCGTTG<br>CCGCTGTTTTGG	987
gp51	AGGAGATATACATACCCGGCATGGAATA<br>CATCGAAAAGG	GCAGCGGCCGCTCCGGAGGCTTCAT<br>CGAACTCTTCTAAATG	1272
gp154	AGGAGATATACATACCC GGC<br>ATGAATAAAATAAAATTAGAAC	GCAGCGGCCGCTCCGGAGGCCTTTT<br>TTGGTTTGGGAC	1140
gp292	AGGAGATATACATACCCGGCATGTCCGT<br>ACATATCACTCG	AGGAGATATACATACCCGGCATGTCC<br>GTACATATCACTCG	705
gp300	AGGAGATATACATACCC GGC<br>ATGAACTACAAAGCATTAG	GCAGCGGCCGCTCCGGAGGCTTTCT<br>TTGCATTCTTATAA	549
gp394	AGGAGATATACATACCCGGCATGTCTCAT<br>CGTAAGGAACT	GCAGCGGCCGCTCCGGAGGCCTTC<br>TTAGGTGCTTTAGATAGG	813
